# Influence of Non-*Saccharomyces* on Wine Chemistry: A Focus on Aroma-Related Compounds

**DOI:** 10.3390/molecules26030644

**Published:** 2021-01-26

**Authors:** Maria Tufariello, Mariagiovanna Fragasso, Joana Pico, Annarita Panighel, Simone Diego Castellarin, Riccardo Flamini, Francesco Grieco

**Affiliations:** 1 CNR–Institute of Sciences of Food Production (ISPA), via Prov. le, Lecce-Monteroni, 73100 Lecce, Italy; 2 Department of the Sciences of Agriculture, Food and Environment, University of Foggia, 71121 Foggia, Italy; mariagiovanna.fragasso@gmail.com; 3 Wine Research Center, Faculty of Land and Food Systems, University of British Columbia, 2205 East Mall, Vancouver, BC V6T 1Z4, Canada; joana.picocarbajo@ubc.ca (J.P.); simone.castellarin@ubc.ca (S.D.C.); 4 Research Centre Viticulture and Enology, Council for Agricultural Research and Economics, 31015 Conegliano, Italy; annarita.panighel@crea.gov.it (A.P.); riccardo.flamini@crea.gov.it (R.F.)

**Keywords:** wine, yeasts, non*-Saccharomyces*, metabolites, alcoholic fermentation, aroma, volatile organic compounds (VOCs), higher alcohols, esters, terpenes

## Abstract

Wine fermentation processes are driven by complex microbial systems, which comprise eukaryotic and prokaryotic microorganisms that participate in several biochemical interactions with the must and wine chemicals and modulate the organoleptic properties of wine. Among these, yeasts play a fundamental role, since they carry out the alcoholic fermentation (AF), converting sugars to ethanol and CO_2_ together with a wide range of volatile organic compounds. The contribution of *Saccharomyces cerevisiae*, the reference organism associated with AF, has been extensively studied. However, in the last decade, selected *non-Saccharomyces* strains received considerable commercial and oenological interest due to their specific pro-technological aptitudes and the positive influence on sensory quality. This review aims to highlight the inter-specific variability within the heterogeneous class of non-*Saccharomyces* in terms of synthesis and release of volatile organic compounds during controlled AF in wine. In particular, we reported findings on the presence of model non-*Saccharomyces* organisms, including *Torulaspora delbrueckii, Hanseniaspora spp,* *Lachancea thermotolerans, Metschnikowia pulcherrima, Pichia* spp. and *Candida zemplinina*, in combination with *S. cerevisiae*. The evidence is discussed from both basic and applicative scientific perspective. In particular, the oenological significance in different kind of wines has been underlined.

## 1. Introduction

Traditional wine-making process is the result of the biological interactions among microorganisms (yeasts, bacteria, fungi) naturally present on the grapes and in the cellar equipment which drives both the alcoholic (AF) and malolactic fermentations (MLF) [1]. The alcoholic fermentation is mainly performed by *Saccharomyces cerevisiae*, referred to as the ‘wine yeast,’ responsible for the conversion of sugars to ethanol and the production/release of numerous secondary metabolites associated with sensory wine characteristics [2]. In addition to *S. cerevisiae*, grape must naturally contain mixed populations of other yeast genera and species, involved, to different extents, in alcoholic fermentation [3]. In spontaneous fermentation process, the initial phase is promoted by the action of a heterogeneous consortium of yeasts belonging to different non-*Saccharomyces* species usually characterised by a low fermentative power, while the final step was distinguished by dominance of *S. cerevisiae* strains [4,5]. In fact, the action of non-*Saccharomyces* is relevant (predominant) during the first stage of alcoholic fermentation until the alcohol level reaches 4% (*v*/*v*), when most *non-Saccharomyces* species can no longer survive [6]. In the past, non-*Saccharomyces* species have been considered ‘bad fermenters’ because of their general low fermentative efficiency, low tolerance to enological additives such as sulfur dioxide and their production of acetic acid. However, in recent years several scientific evidences have encouraged researchers and producers to reconsider the action and contribution of the *non-Saccharomyces* species to the improvement of wine processing and product quality, especially in synergy with *S. cerevisiae* [2,3,7,8,9]. However, in the past, non-*Saccharomyces* fermentations have been generally associated with high volatile acidity, ethyl acetate production, *off*-flavours and wine spoilage. Emerging evidence has demonstrated that the exclusive use of *S. cerevisiae* could result in a sensorial flattening of wines when compared with distinctive aromatic features of wines produced by spontaneous fermentation driven by the above-described yeast consortium [9,10]. Although the oenological potential of *Saccharomyces* strains has been deeply investigated, there is scarce information related to the impact of different non-*Saccharomyces* genera/species on the aromatic profile of wines [3,4,10,11,12,13,14,15,16].

Recent studies have shown that non-*Saccharomyces* yeasts have different oenological properties compared to those of *S. cerevisiae,* which can be used to solve specific technological issues (e.g., reduce volatile acidity, biocontrol strategies to prevent spoilage) in order to modulate and enrich sensory properties of wines by playing a role in releasing volatile compounds from non-volatile precursors [2,11,17,18,19]. The use of non-*Saccharomyces* species as part of mixed starters together with *S. cerevisiae*, and eventually also with malolactic bacteria, has been suggested as a way to mimic spontaneous fermentations and enhance wine complexity [8,9,13,17,20]. Additionally, the use of these mixed yeast starters can avoid the risks of fermentative stuck [6,8,21,22]. Promising research has been conducted on the fermentation performances of various strains, on their ability to control wine spoilage and to improve aroma and complexity of wines. The different levels of enzymatic activities and the different abilities to produce secondary metabolites that characterise non-*Saccharomyces* yeasts, are some of the aspects to be considered among criteria for the strain selection and starter cultures design [13,20,23,24]. 

The market demand for wines with lower alcohol content, more complex sensory profile, low volatile acidity, pronounced antioxidant activity and a minor impact of exogenous chemistry has driven an increasing interest of researchers and stakeholders in a better understanding of properties and innovation on non-*Saccharomyces* in the oenological sector [25].”This review aims to summarise the current knowledge about the role of non-*Saccharomyces* yeasts of oenological interest in enhancing the wine quality through the production of specific volatile compounds during the vinification process.

## 2. The Main Chemical Categories behind Wine Aroma

Undoubtedly, *flavour* is one of the most important characteristics that contribute to the quality of wine. Several hundreds of different compounds, with concentrations ranging from 100 mg/L to 0.1 μg/L contribute to the composition of wine aroma [20,21]. The final aroma of wine is known as a combination of the volatile compounds originating from the grapes (varietal or primary aroma), the yeast and bacterial metabolism (the fermentation or secondary aroma) as well as the post-fermentative wine-making practices, including ageing processes (tertiary aroma) [21,22]. The production of active compounds of primary wine aroma takes place in the exocarp of the grape berry and its final concentration in wine is mainly influenced by the vine variety and subsidiary by the state of ripeness and the agronomic and oenological practices, as shown for several French (*Semmillon, Merlot, Chardonnay*), Italian (*Corvina, Negroamaro, Primitivo*) and Spanish (*Tempranillo*) grape cultivars [23,24,25,26,27]. On the one hand, the grape-derived aroma compounds play a key role in the expression of distinctive aroma attributes in the corresponding wine. On the other hand, during alcoholic fermentation, wine yeasts, including non-*Saccharomyces*, are able to influence wine aroma through different pathways of *de novo* biosynthesis of specific compounds (secondary aromas), but they also influence the chemistry of primary compounds, through specific enzymatic activities. Table 1 provides a brief introduction of the main classes of volatile organic compounds affected by yeasts metabolism and involved in shaping wine aroma. 

**Figure 1:**
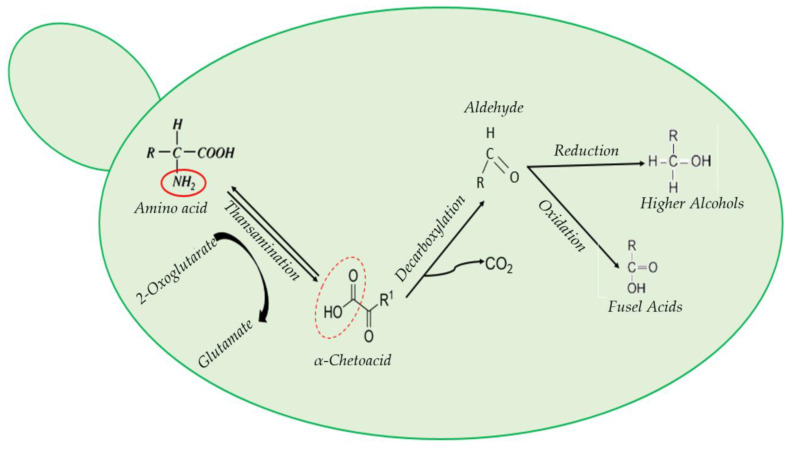
Amino acids (branched-chain amino acids, leucine, isoleucine and valine, aromatic amino acids, phenylalanine, tyrosine and tryptophan, or methionine) are converted in the Ehrlich pathway to fusel alcohol or fusel acids in a three-step process.

Higher alcohols and aldehydes and their associated esters and acids are commonly generated through the Ehrlich pathway, in which the yeasts use some of the amino acids to produce these aroma compounds (Figure 1). 

Some of the amino acids involved in this reaction are non-polar branched-chain amino acids (valine, leucine and isoleucine) and these are catabolised into isobutanol, isoamyl alcohol and amyl alcohol [50]. The Ehrlich’s reaction is based on two steps: firstly, a transamination reaction in which the amino group from the amino acid is transferred to an α-ketoglutarate to form an α-keto acid and glutamate [50,54]. Secondly, α-keto acid is decarboxylated into an aldehyde [55] and then finally due to redox status of the yeast cell, the aldehyde can be reduced to its respective higher alcohol or can be oxidised into a volatile carboxylic acid. The ability to produce higher alcohols is strictly strain-dependent, and this feature can be used as a determining character in yeast selection for industrial purposes [19,21]. 

**Figure 2:**
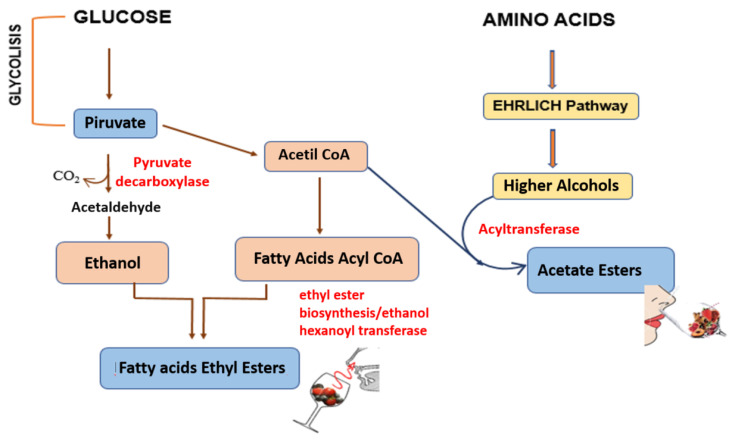
Acetate ester and ethyl ester biosynthesis.

Regarding the production of the esters, in wine there are two groups of esters: ethyl esters and acetate esters, both responsible of the fruity notes of the wine. The biosynthesis of these esters follows two ways: (1) the direct, enzyme-free formation that is an equilibrium reaction between an alcohol and an acid; (2) the enzymatic reaction that involves different enzymes (Figure 2). 

The enzymatic formation of ethyl esters required an initial activation of the acid by combining it with coenzyme A (CoA) before reacting with the alcohol to form an ester [54]. The coenzyme donor can either be acetyl-CoA (formed from pyruvate) or any of a range of acyl-CoA compounds formed by the enzyme acyl-CoA synthetase [50,54]. Thus, ethyl esters of fatty acids (such as ethyl butanoate, ethyl hexanoate, ethyl octanoate) are formed from the ethanolysis of fatty acyl-CoA (an intermediate metabolite of fatty acid metabolism) [56,57]. The other group of esters, the acetate esters (such as isoamyl acetate, propyl acetate, hexyl acetate, phenethyl acetate), are the result of the reaction of acetyl-CoA with alcohols that are formed from the degradation of amino acids, carbohydrates and lipids [45,46], which is catalysed the enzyme alcohol acetyltransferase (AAT).

The pool of grape-derived volatile compounds in wine includes potent odorants such as C13-norisoprenoids and methoxypyrazines [26,57,58]. In the grapes, the synthesis of methoxypyrazines is linked to the amino acid metabolism, these pyrazine derivatives being responsible for vegetal, green and herbaceous notes in specific vine cultivars [59]. The formation of C13-norisoprenoids in grape berry involves carotenoid breakdown. It was initially proposed that carotenoids could be degraded by chemical, photochemical, and oxidase-coupled mechanisms, but recent studies supported the hypothesis of the involvement of a region-specific oxygenase (CCD) in the formation of C13-norisoprenoids [60]. The C13-norisoprenoids, associated with floral or fruity notes, constitute an essential part of the volatile compounds of non-floral grapes, such as *Cabernet Sauvignon, Syrah*, *Sauvignon Blanc* and *Pinot noir* [61]. 

Monoterpenes (C10) and sesquiterpenes (C15) are also essential and potent aromas from the grape that strongly affect the aroma of the corresponding wine. They are lower molecular weight molecules from the terpenoid family, composed of two (monoterpenes) and three (sesquiterpenes) isoprene units, respectively. Two independent pathways produce terpenes in grapes: 1) the plastidial 2C-methyl-d-erythritol-4-phosphate (MEP) pathway, which is the predominant pathway for monoterpenes (C10), and 2) the cytosolic mevalonate (MVA) pathway, which is the primary pathway for sesquiterpenes [62]. They are by-products of some enzymatic transformations such as oxidation, reduction and dehydration and floral, muscatel, or fruity aromas are characteristic molecules belonging to terpene family [63]. These compounds are synthesised and stored in the berry as glycosides or free, and their concentrations in wine depend on various factors, including cultivar, region and wine-making techniques [26,62,63]. Concerning terpenoids, in grape berries and corresponding wines, approximately twenty-two different molecules have been identified in grapes and wine [56]. Among them, five monoterpenoid alcohols, namely linalool, geraniol, nerol, citronellol, and α-terpineol, are the most abundant and having low odour threshold that significantly contribute to the wine aroma with floral notes. 

**Figure 3:**
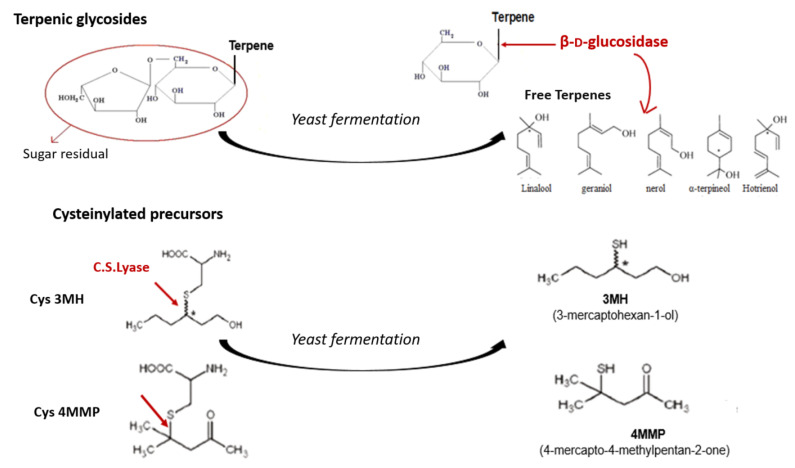
Production of varietal aroma compounds during fermentation process. Monoterpenes and volatile thiols are detected in grapes as odorless precursors after the release of primary aroma compounds due to yeast enzymatic activity. A two-step enzymatic reaction is responsible for the enzymatic hydrolysis of the diglycosides and to production of free terpene. Volatile thiols are generated from the odourless cysteinylated precursors cysteine-3-mercaptohexan-1-ol (Cys-3MH) and cysteine-4-mercapto-4-methylpentan-2-one (Cys-4MMP) by the action of carbon-sulfur-lyases (C.D.Lyase).

Noteworthy, the terpenols and C13-norisoprenoids are present in grapes in free or bound form, the latter being considered as the ‘aroma precursors’. Bound terpenols and C13-norisoprenoids are linked to sugar molecules, and the generation of the corresponding aglycone occurs after hydrolysis due to the action of yeast-secreted enzymes, as it is shown in Figure 3. 

The enzymatic hydrolysis of glycosylated terpenes occurs in two-steps (Figure 3). During the first step, the monoterpenyl-β-d-glucosides release through the action, depending on the conjugate, of α-l-arabinofuranosidase, an α-l-rhamnosidase or a β-d-apiosidase. In the second step, monoterpenes are liberated by the action of a β-d-glucosidase. The species-specific presence of β-d-glucosidase activity in non-*Saccharomyces* yeasts is well reported in literature, being the reason why different genera/species may have a different oenological impact. [57,58]. For example, the enzymes from *Hanseniaspora sp*. were more efficient than the enzymes of other yeast species in releasing desirable aromas during an early stage of alcoholic fermentation, while the β-d-glucosidase secreted by *P. anomala* was more efficient during the final stage of the vinification process [64]. 

## 3. Grape and Wine Aroma Composition

The main aroma compounds in grape, belonging to the chemical classes of monoterpenes, C13-norisoprenoids and benzenoid compounds, are mainly contained in the berry skin in both free and glycoside forms. Their profiles in wines reflects the grape variety used, even if environmental variables and agricultural practices may, to some extent, influence their contents in grape [65]. In wine-making, they are transferred to the fermenting must with an intensity also depending on the process used.

**Figure 4:**
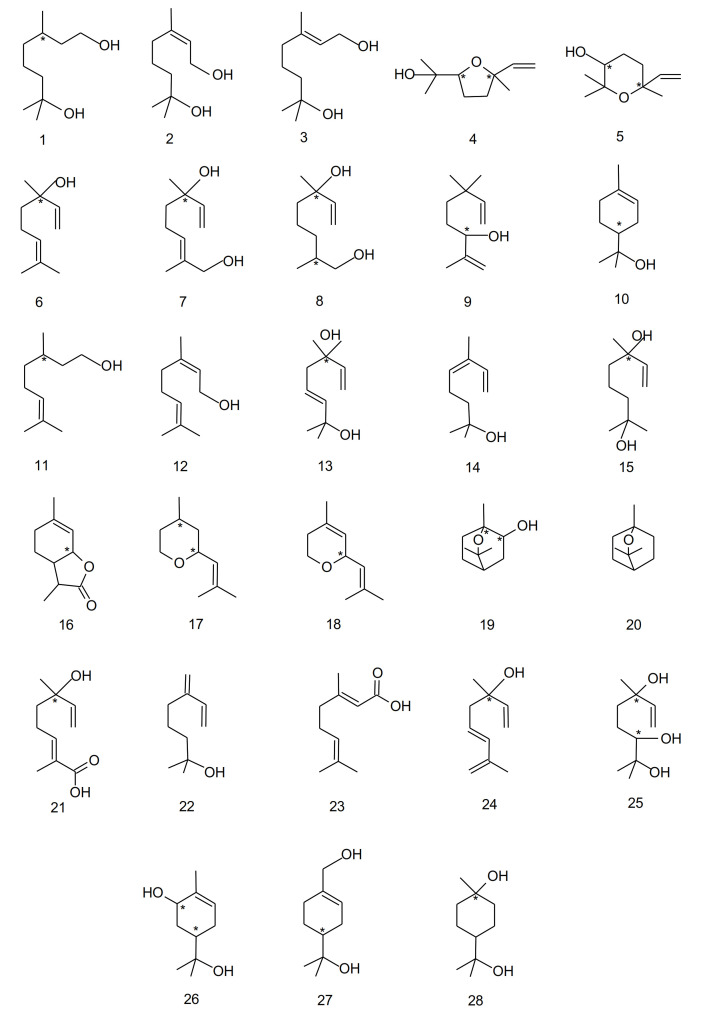
Main monotepenes in grape and wine. (**1**) hydroxycitronellol (3,7-dimethyloctane-1,7-diol); (**2**) 7-hydroxynerol (*Z*-3,6-dimethyl-2-octene-1,7-diol); (**3**) 7-hydroxygeraniol (*E*-3,6-dimethyl-2-octene-1,7-diol); (**4**) *cis* and *trans* linalool oxide (5-ethenyltetrahydro-,5-trimethyl-2-furanmethanol) (furanic form); (**5**) *cis* and *trans* linalool oxide (2,2,6-trimethyl-6-vinyltetrahydro-2*H*-pyran-3-ol) (pyranic form); (**6**) linalool (3,7-dimethyl-1,6-octadien-3-ol); (**7**) *cis* and *trans* 8-hydroxylinalool (*Z* and *E* 2,6-dimethyl-2,7-octadiene-1,6-diol); (**8**) 8-hydroxydihydrolinalool (2,6-dimethyl-7-octene-1,6-diol); (**9**) Ho-diendiol II (3,7-dimethyl-1,7-octadiene-3,6-diol); (**10**) α-terpineol (α,α,4-trimethyl-3-cycloexene-1-methanol); (**11**) citronellol (3,7-dimethyl-6-octen-1-ol); (**12**) nerol (*Z*), geraniol (*E*) (3,7-dimethyl-2,6-octadien-1-ol); (**13**) Ho-diendiol I (3,7-dimethyl-1,5-octadiene-3,7-diol); (**14**) *Z* and *E* ocimenol (2,6-dimethyl-5,7-octen-2-ol); (**15**) endiol (3,7-dimethyl-1-octene-3,7-diol); (**16**) wine-lactone; (**17**) *cis* and *trans* rose oxide; (**18**) neroloxide; (**19**) 2-exo-hydroxy-1,8-cineole; (**20**) 1,8-cineole; (**21**) *E*-2,6-dimethyl-6-hydroxyocta-2,7-dienoic acid; (22) myrcenol (2-methyl-6-methylene-7-octen-2-ol); (23) *E*-geranic acid (3,7-dimethyl-2,6-octadienoic acid); (**24**) hotrienol [(*E*)-3,7-dimethylocta-1,5,7-trien-3-ol]; (**25**) 2,6-dimethyl-7-octene-2,3,6-triol; (**26**) *E* and *Z* sobrerol or *p*-menthenediol II (*p*-menth-1-ene-6,8-diol); (**27**) *p*-menthenediol I (*p*-menth-1-ene-7,8-diol); (**28**) *cis* and *trans* 1,8-terpin.

Monoterpenols confer the characteristic floral or citrus notes to aromatic and semi-aromatic grape varieties, such as *Muscat* and *Malvasie*, *Gewürztraminer*, *Riesling*, *Glera* are mono- and di-hydroxylated compounds and ethers [66,67,68,69,70,71]. Structures of the main terpenols identified in grape are reported in Figure 4.

**Figure 5:**
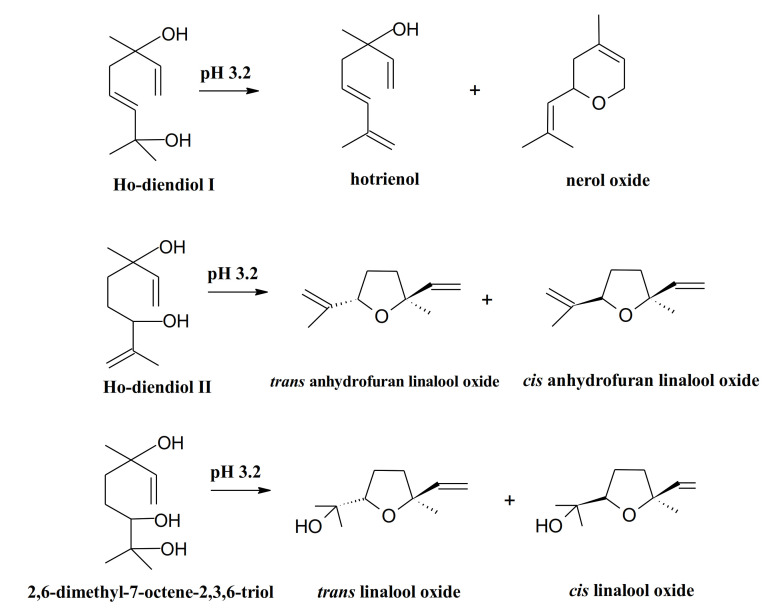
Acid catalyzed hydrolysis of non-floral diols and 2,6-dimethyl-7-octene-2,3,6-triol.

The monoterpene profile change during wine-making and wine ageing because of acid-catalysed reactions promoted by acidic pH of wine [68]. Rearrangement of geraniol, nerol and α-terpineol induces formation of linalool, -terpineol and 1,8-terpins and of non-floral diols and 2,6-dimethyl-7-octene-2,3,6-triol lead the formation aromatic compounds such as neroloxide, roseoxide, anhydrofuranes and anhydropyranes [67,72]. Enantiospecific reduction of geraniol and nerol performed by the yeasts promotes formation of (*R*)-(+)-citronellol [73] and rearrangement of (*E*)-2,6-dimethyl-6-hydroxyocta-2,7-dienoic acid the formation of highly odorant wine-lactone (3a,4,5,7a-tetrahydro-3,6-dimethylbenzofuran-2(3*H*)-one) [6,14]. A scheme of acid-catalysed reactions of monoterpenols is shown in Figure 5.

**Scheme 1:**
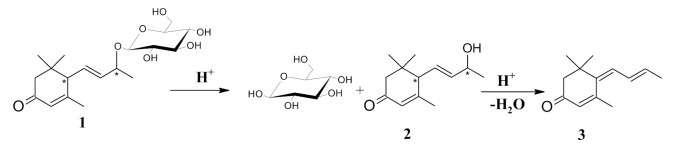
Formation of megastigmatrienone (**3**) in wine from glycoside 3-oxo-α-ionol precursor occurring during ageing. Asymmetric carbon atoms in the molecule are designated by asterisks.

**Figure 6:**
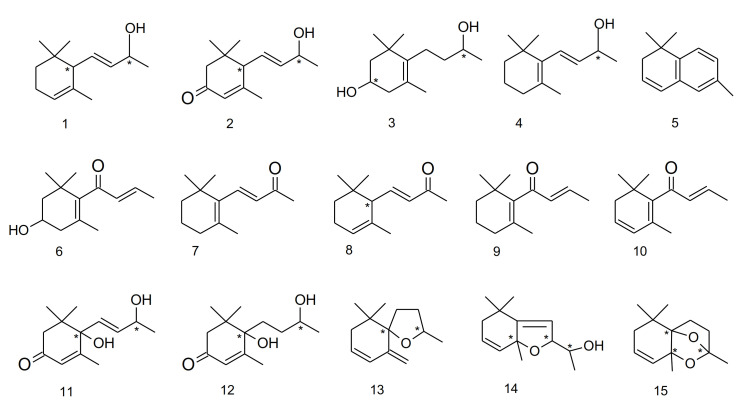
Main norisoprenoid compounds identified in grape and wine: (**1**) α-ionol; (**2**) 3-oxo-α-ionol; (**3**) 3-hydroxy-7,8-dihydro-β-ionol; (**4**) β-ionol; (**5**) TDN (1,1,6-trimethyl-1,2-dihydronaphthalene); (**6**) 3-hydroxy-β-damascone; (**7**) β-ionone; (**8**) α-ionone; (**9**) β-damascone; (**10**) β-damascenone; (**11**) vomifoliol; (**12**) dihydrovomifoliol; (**13**) vitispiranes (spiro [4.5]-2,10,10-trimethyl-6-methylene-1-oxa-7-decene); (**14**) actinidols; (**15**) Riesling acetal (2,2,6-tetramethyl-7,11-dioxatricyclo[6.2.1.0^1,6^]undec-4-ene). Asymmetric carbon atoms in the molecule are designated by asterisks.

C13-noisoprenoids play an important role in the typical aroma of some white wines and grape varieties, such as *Chardonnay*, *Semillon*, *Sauvignon blanc, Torbato*, and red wines such as *Shiraz*, *Grenache*, *Merlot* and *Cabernet Sauvignon* [74,75] and in the aroma of aged wines [74,76,77,78,79,80]. 1,1,6-trimethyl-1,2-dihydronaphthalene (TDN), vitispiranes, actinidols and β-damascenone confer to the wine kerosene, resinous/eucalyptus-like, woody and rose-like scents, respectively. E.g., the tobacco aroma typical of wines produced by using *Sangiovese* grape (e.g., *Brunello di Montalcino* wines) and *Valpolicella* wines is related to β-damascenone, 3-oxo-α-ionol, (*E*)-1-(2,3,6-trimethylphenyl)-buta-1,3-diene (TPB), and some megastigmatrienone isomers. Latter compounds are formed by following a hydrolysis + rearrangement mechanism of 3-oxo-α-ionol glycoside which may occur during ageing (Scheme 1) [81,82]. Structures of the main norisoprenoids found in wine are reported in Figure 6.

Flavouring benzenoid compounds present in non-barrel-aged wines are mainly derived from grape. Main are vanillin (vanilla note), ethyl-vanillate (flowery), methyl-vanillate (dry herbs), β-phenylethanol (rose note) [83], zingerone (sweet, fruity, cooked pears notes) [84], and methyl salicylate (floral note, slightly balsamic, tending to cinnamon-chestnut honey) [77,85,86,87].

Anyway, the higher amounts of benzenoid volatile compounds present in aged wines are due to release by wood barrels as they are formed by lignin degradation during the toasting process of wood staves [88,89,90], so as furan derivatives are formed by thermal degradation of carbohydrates (almond, roasted/toasty, and caramel-like notes) [91,92,93,94].

3-Alkyl-2-methoxypyrazines present in grape skin, pulp and bunch stems contribute to wine aroma with their characteristic vegetative, herbaceous, bell pepper or earthy notes, in particular in *Cabernet Sauvignon*, *Sauvignon blanc*, *Semillon* wines [95,96,97,98,99]. The level of isobutylmethoxypyrazine in wine can be 10-fold its sensory threshold, sec-butylmethoxypyrazine and isopropylmethoxypyrazine are normally present in contents close to their sensory thresholds, 2-methoxy-3-isobutylpyrazine, 2-methoxy-3-*sec*-butylpyrazine and 2-methoxy-3-isopropylpyrazine can impact wine aroma because are characterised by particularly low sensory thresholds (1–2 ng/L in water) [95,96,97,98,99,100].

In *Shiraz* grape, rotundone ((3*S*,5*R*,8*S*)-5-isopropenyl-3,8-dimethyl-3,4,5,6,7,8-hexahydro-1(2*H*)-azulenone) was identified as the characteristic compound which confers pepper-aroma [101]. In addition, many sulphur-compounds can be present in wines. They are formed both by enzymatic processes of the yeasts during fermentation and chemical, photochemical and thermal reactions occurring in wine-making and wine ageing [102]. They belong to the chemical classes of thiols, sulphides, thioesters, and heterocyclic compounds, and many of them can play a detrimental effect on the wine aroma [102,103], e.g., *trans*-2-methylthiophan-3-ol and 4-methylthiobutan-l-ol confer garlic odour to the wine, onion odour is associated to 2-methyltetrahydrothiophenone, burnt, cabbage is associated to methionol and cauliflower to 2-(methylthio)-ethanol [104].

3-Mercaptohexan-1-ol (3-MH) and 4-methyl-4-mercaptopentan-2-one (4-MMP) are compounds with tropical fruit-like scent characteristic of *Sauvignon blanc* wines. In particular, 4-MMP at ppt levels confers the box-tree like aroma typical of *Sauvignon blanc* wines. In grape, they are present as S-cysteine and glutathionyl conjugates [105] and in wines also 3-mercaptohexyl acetate (3-MHA) can be present [106,107]. Other sulphur-compounds which can be present in wines are ethyl mercaptan (EtSH), dimethyl sulphide (DMS, grassy/truffle-like), diethyl sulphide (DES), dimethyl disulphide (DMDS), diethyl disulphide (DEDS), methyl thioacetate (MTA), ethyl thioacetate (ETA), 2-mercaptoethanol (ME), 2-(methylthio)-1-ethanol (MTE), 3-(methylthio)-1-propanol (MTP), 4-(methylthio)-1-butanol (MTB, earthy-like scent), benzothiazole (BT) and 5-(2-hydroxyethyl)-4-methylthiazole (HMT). DMS, MTB, MTE and BT are characteristic of *Merlot* wines [108].

2-Methyl-3-furanthiol (MF, a very odoriferous compound with 0.4–1.0 ppt odour threshold) and bis(2-hydroxyethyl) disulfide were identified in wines from *V. vinifera* and *V. labrusca* grapes, respectively [109,110]. The latter is a precursor of H_2_S and 2-mercaptoethanol characterised by strong rotten eggs and unpleasant poultry-like odour, respectively.

Yeasts and bacteria fermentation (e.g., malolactic fermentation, MLF) produce also many carbonyl compounds which play an important role in determining the sensorial characteristics of wine. MLF is a biological process carried out by lactic bacteria often at industrial-scale aimed to improve the organoleptic characteristics and confer microbiological stability to the wines [111]. This process induces profound changes in the profile of carbonyl compounds by conferring complexity to the wine aroma [112,113]. Aliphatic aldehydes, such as hexanal, (*E*)-2-hexenal, (*E*)-2-heptenal, octanal, and (*E*)-2-octenal, confer herbaceous odour to the wine [114,115], decanal and (*E*)-2-nonenal are associated with ‘sawdust’ or ‘plank’ odour [116], diacetyl is regarded to add complexity to wine but if present in relevant concentration (higher 5 mg/L) it can be overpowering and to confer a distinct butter-like undesirable note. MLF carried out by *Oenococcus oeni* produces glyoxal, methylglyoxal and hydroxypropandial [117,118,119] but these compounds are in general characterized by very low odour thresholds [117,118]. During barrel storage wine can undergo attack by slow-growing species, such as *Brettanomyces bruxellensis*, *B. anomalus*, *S. bailli* and certain genera of lactic bacteria. This phenomenon can be due to difficulty to clean and sterilise wooden barrels [120]. 

**Scheme 2:**
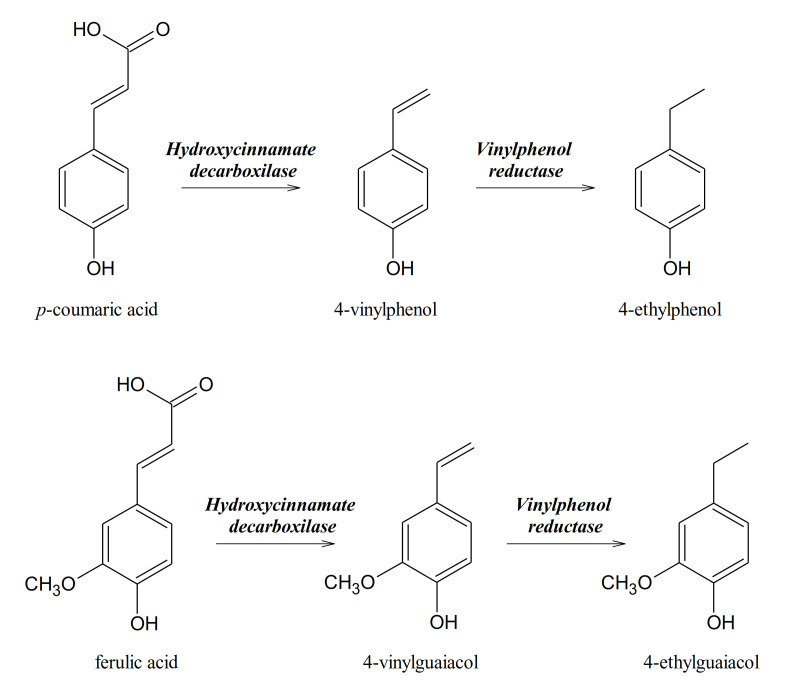
Formation of ethylphenols in wine.

Finally, several volatile phenols originated by degradation of ferulic acid, *p*-coumaric acid and caffeic acid by action of hydroxycinnamate decarboxylase and reductase enzymes present in some species (e.g., *B. bruxellensis*, *D. anomala*, *Pichia guillermondii*, *Candida versatilis*, *C. halophila* and *C. mannitofaciens*) can be present in wines [121,122,123,124,125]. Small amounts of these compounds can also be produced by the activity of yeasts and lactic and acid bacteria under particular growth conditions [126,127,128]. A scheme of ethylphenols formation is shown in Scheme 2.

Compounds such as 4-vinylphenol (4-VP), 4-vinylguaiacol (4-VG), 4-ethylphenol (4-EP) and 4-ethylguaiacol (4-EG) can greatly influence wine aroma. The odour thresholds in wine reported for 4-EP and 4-EG are 440 and 33 g/L, respectively [75,81,82,83,123,129,130]. White wines can contain vinylphenols in concentration up to hundreds µg/L, but usually they lack of ethylphenols which instead in red wines can reach several mg/L [75,82]. In red wines, the disagreeable odour described as “phenolic”, “leather”, “horse sweat”, “stable” or “varnish” is due to the presence of 4-EP. In general, vinylphenols in wine are classified among “off-flavours” and described as phenolic, medicinal, pharmaceutical, smoky, spicy and clove-like [131]. 4-VP is deemed to negatively affect and mask the fruity scent of white wines [132] conferring odours resembling “band-aid” and gouache [133], instead 4-VG contributes to the floral aroma of *Chardonnay* wines [134] and to the spicy note in *Gewürztraminer* wines [135].

Ultra-High Performance Liquid Chromatography/High-Resolution Mass Spectrometry (HPLC/HRMS) methods, which have been recently developed, allow analysis of glycosidic aroma precursors directly in their intact form [136,137]. This approach allows to study both the molecule aglycone and the sugar residue, so it was possible to characterize the structures of many grape monoterpenes glycosides including several monoterpendiols pentosyl-hexoside (*p*-menthenediol I, furan and pyran linalool oxides, 7-hydroxygeraniol and 7-hydroxynerol, diendiol I, *cis*/*trans* 8-hydroxylinalool), several monoterpenols (α-terpineol, linalool, nerol, geraniol, citronellol) in their glucoside, pentosyl-hexoside, rhamnosyl-hexoside, hexose-deoxyhexose and malonyl-glucoside, dihydromonoterpenetetraols hexose and pentose−hexose forms, some dihydromonoterpenetriols hexose and pentose−hexose, one dihydromonoterpenetriol deoxyhexose−hexose, some monoterpenetriols pentose−hexose, one monoterpenetriol deoxyhexose−hexose, some dehydromonoterpenetriols hexose and dihydromonoterpenediols pentose−hexose, one monoterpenediol deoxyhexose−hexose, and some monoterpenols deoxyhexose−hexose [137,138]. Moreover, the structures of some hydroxynorisoprenoids hexose and hexose−hexose, some hexose, hexose-hexose and pentose−hexose norisoprenoids and sesquiterpenols hexose−pentose, were identified [139]. Knowledge of the complete structure of the precursor can be useful to select specific yeast strains and enzymes to perform liberation of target aroma compounds.

## 4. Impact of Non-*Saccharomyces* Species on Wine Aroma

Generally, wines are distinguished by the different concentrations of volatile molecules influenced by the type of yeast used and the fermentation conditions. Thus, the biosynthesis of these compounds such as alcohols, esters, acids or aldehydes is strongly species- and strain-dependent and the positive or negative impact of these molecules on the wine aroma is influenced by their concentrations. For these reasons, an increasing number of investigations have explored the contribution provided by several non-*Saccharomyces* yeast genera/species. The focus of this review is to encompass and compare the outcomes of several studies reporting the fermentation performances of specific non-*Saccharomyces* genera/species (selected based on their oenological relevance): *Torulaspora delbrueckii, Hanseniaspora spp, Lachancea thermotolerans, Metschnikowia pulcherrima, Pichia* spp. and *Candida zemplinina*. 

### 4.1. Torulaspora delbrueckii

*Torulaspora delbrueckii* is one of the most investigated non-*Saccharomyces* yeast, in reason of its relatively higher alcohol tolerance [9–10% (*v/v*)], especially if compared to others non-*Saccharomyces* genera/species [14]. *Torulaspora delbrueckii* demonstrated the ability to produce low levels of acetic acid in mixed formulation with a *S. cerevisiae* strain [14]. Some authors reported significant production of linalool in Muscat wine due to enzymatic activity of *T. delbrueckii* [36]. Among terpenes, linalool is positively associated with floral aroma with spicy lemon and tones in wine [140]. *Soave* and *Chardonnay* wines with fermentations driven by *T. delbrueckii* and *S. cerevisiae* in sequential inoculum showed a significant decrease of some volatiles including 2-phenylethanol (rose odour note), isoamyl acetate (banana note), C_4_–C_10_ volatile acids and vinylphenols, thus producing wines denoted by a greater aromatic intensity and complexity than those resulting from a monoculture fermentation [141]. Recently, Zhang and co-workers [29] have studied the effect of sequential and co-inoculation of *S. cerevisiae* (indigenous and commercial) together with *T. delbrueckii*, in the volatile composition of *Cabernet Sauvignon* wine. These authors have demonstrated that in co-inoculation condition, mixed starters produced higher values of 3-methyl-1-butanol, 2-phenylethanol, ethyl butanoate and ethyl decanoate and also reduced volatile acids concentration. However, the sequential inoculation promoted a significant enhancement of aroma due to an increase of more esters, phenylethyl alcohol and phenylacetaldehyde, intensifying the fruity, flowery, and sweet character of the final wine. Renault et al. [39] described similar results by evaluating the specific production of esters by this microorganism. The authors identified ethyl propanoate, ethyl isobutanoate and ethyl dihydrocinnamate as markers of the activity of *T. delbrueckii* and they also associated an increase of isobutyl acetate and isoamyl acetate concentrations with the interactions between *T. delbrueckii* and *S. cerevisiae*.

The fermentative performance of *T. delbrueckii* was also investigated during Amarone must fermentation [40]. Compared to control fermentation process driven by *S. cerevisiae,* the presence of *T. delbrueckii* yielded an increase of some alcohols including benzyl alcohol and phenylethanol, esters and lactones. In particular, the benzyl alcohol increase was strictly linked to the enzymatic activity promoted by *T. delbrueckii* on benzaldehyde, its glycoside precursor [142]. Likewise, the increase of phenylethanol in *Amarone* wine also appeared to depend strictly on β-glucosidase activity and the level of this molecule was 4-fold higher than in the control wine. In conclusion, the authors affirmed that *T. delbrueckii* has a higher β-glucosidase activity than *S. cerevisiae* [40]. This enzymatic activity allows *T. delbrueckii* to positively affect the sensorial profile, releasing varietal terpenes from odourless precursors or influencing alcohols production. 

### 4.2. Hanseniaspora spp.

*Hanseniaspora* is a genus that has been extensively studied in the last years because of the ability of its several species to improve the aromatic complexity of wines in mixed inoculum with *S. cerevisiae*. Mestre et al. [143] studied twenty-eight *H. uvarum* isolates and found some interesting properties, such as their ability to grow at high sugar, ethanol and SO_2_ contents, to produce high concentrations of low acetic acid and to release proteolytic enzymes. Regarding the ethanol production, *Hanseniaspora* species are considered low producers because it requires more than 19 g/L of consumed sugar to produce 1% *v/v* of ethanol [144]. Concerning the volatile acidity production, *Hanseniaspora* spp. have been traditionally described as producers of excessive amounts of acetic acid [35]. However, several investigations have demonstrated that the use of mixed the starter cultures of *S. cerevisiae* and *H. uvarum* strains resulted in a reduction of acetic acid concentrations, even lower than those produced by the pure culture of *S. cerevisiae* [7,145].

Selected *H. uvarum* strains were used in mixed fermentation to improve the wine aroma in *Sauvignon Blanc* white wine from South Africa [7,10] and *Negroamaro* red wine from Southern Italy [7,10]. In *Negroamaro* wine, the partnership between *H. uvarum* and *S. cerevisiae* led to the increase of some esters including phenylethyl acetate and ethyl octanoate (fruity odour notes) and the decrease in some higher alcohols like phenylethanol (floral odour notes) and hexanoic and octanoic acids. *Hanseniaspora* *uvarum* yeast is considered a high acetate ester producer [37]. 

Due to its esterase activity, *H. uvarum* can positively modulate the flavour of wine through the increase of some ethyl and acetate esters, including phenylethyl acetate, or by significant decrease of phenylethanol and 3-methyl-1-butanol [7,145]. *H. uvarum* strains are characterised by the other important enzymatic activities such as β-glucosidase*,* lipases and proteases, all capable of contributing to the improvement of the wine organoleptic quality [146]. Hu et al. [33] documented that the floral and fruity qualities *in Cabernet Sauvignon* and *Ecolly* wines were characteristically modified by the inoculation of *H. uvarum*, which modulated the final amounts of specific fermentative and varietal volatile compounds. *H. uvarum* also showed a higher β-glucosidase activity than *S. cerevisiae*, explaining the increase of terpenes and norisoprenoids levels in wines in a varietal way [33]. Moreover, Mendes Ferreira et al. [34] showed that in *Muscat* grape juice, *H. uvarum* was able to better release linalool, geraniol, nerol, α-terpineol, *o*-cimenol and citronellol, improving varietal character of the wine.

### 4.3. Lachancea thermotolerans

*Lachancea thermotolerans* is a ubiquitous yeast that can be commonly found on grape berry surface [147]. *Lachancea thermotolerans* is known for its ability to produce wines with a low volatile acidity. For this reason, there is an interest in using them in mixed starter cultures (with *S. cerevisiae* or other non-*Saccharomyces* species) [8,12]. The fermentative activity of this yeast species is characterised by an increased in lactic acid production, which allows the wine pH to be naturally controlled, avoiding the addition of exogenous acids such as tartaric acid [148,149]. Moreover, the ability of *L. thermotolerans* to use sugars for lactic acid production can contribute to decreasing the ethanol level during fermentation [12]. This behaviour was confirmed by a study on the impact of *L. thermotolerans* on Emir wine fermentation [42]. In this study the authors observed that when *L. thermotolerans* was mixed and sequentially inoculated with *S. cerevisiae*, there was an increase of the total acidity content followed by decrease of volatile acidity (to a final level below 0.8 g acetic acid/L wine). In addition, the authors reported an increase of esters, which adds notes of fruitiness, followed by a decrease of acetaldehyde and higher alcohols. Besides, the contribution made by this microorganism to the development of varietal aromas from odourless precursors has been investigated, showing that it promoted the formation of higher alcohols esters and reduced volatile phenols [149]. 

*Lachancea thermotolerans* shows β-d-glucosidase and carbon-sulfur lyase activities, it being enzymes involved in the release of aroma compounds from must volatile precursors [150]. Selected strains showed to be able to enhance the contents of nerol, terpinen-4-ol, 2-phenylethanol, phenethyl propionate, ethyl salicylate, methyl salicylate and 3-methylthio-1-propanol in Syrah and Sauvignon Blanc wines [12]. Several studies also highlighted the *L. thermotolerans* ability to produce relevant glycerol amounts during fermentation, in particular when sequential inoculation with *S. cerevisiae* occurred [151]. Indeed, a vinification carried out in the presence of *L. thermotolerans* resulted in significant decreases in acetic acid concentration and enhancement of total acidity, glycerol and 2-phenylethanol amounts [52]. These features also make autochthonous strains of *L. thermotolerans* a right starter candidate to improve sensory quality and enhance the typicality of regional wines by linking the wine characteristics specifically to the ‘terroir’ or environment [152]. Moreover, the ability of *L. thermotolerans* to act as an acidifying agent (lactic acid producer) is of increasing interest, as global climate change and variations in viticulture and oenology practices have resulted in a trend towards the reduction of the total organic acids concentration [153].

### 4.4. Metschnikowia pulcherrima

*Metschnikowia pulcherrima* is a non-*Saccharomyces* yeast that shows potential to biocontrol spoilage yeasts and displays important enzymatic activities, such as pectinase, protease, glucanase, lichenase, β-glucosidase, cellulase, xylanase, amylase, sulfite reductase, lipase and β-lyase [10,154]. As a consequence of their enzymatic potential, it is an excellent partner of *S. cerevisiae* in mixed starter culture formulation. It has been demonstrated that, through its proteolytic activity, it is able to promote amino acids degradation, thus favouring a better availability of nitrogen for the growth of *S. cerevisiae* [4,11]. Shifting to the effects on the aroma-related compounds, enzymes produced by *M. pulcherrima* modulate the release of terpenes [4,11]. Initially, by α-L-arabinosidases cleavage the terminal arabinose and the corresponding β-d-glycosides are released. Subsequently, the release of terpene occurs after the action of β-d-glucosidases [4,11]. *M. pulcherrima* strains also contribute to volatile thiol release in wines. This action is regulated by β-lyase activity and mainly occurs during the pre-fermentation stage [150]. The positive impact of *M. pulcherrima* on esters production was also confirmed by several studies [52,54]. Additionally, the co-inoculation of *M. pulcherrima* and *S. cerevisiae* has been reported to improve 4-methyl-4-sulfanylpentan-2-one and 2-phenylethanol in *Verdejo* wine and higher levels of acetate esters and β-damascenone in *Vidal Blanc* wine [29,43]. However, Varela et al. [44] demonstrated that *M. pulcherrima* strains produced ethyl acetate in concentrations that negatively affected the wine aroma, but when it was used in co-inoculum with *S. cerevisiae* they produce 2-phenyl ethanol and 2-phenylethyl acetate (responsible for floral and fruity odour notes).

### 4.5. Candida zemplinina

*Candida zemplinina* (synonym *Starmerella bacillaris)* is a non-*Saccharomyces* yeast that detains important oenological features including the ability to grow in very sweet musts, to tolerate high alcohol levels, and to produce relevant amount of glycerol [45,155]. Additionally, this yeast species is able to produce wines with reduced ethanol concentration, this being explained by the production of secondary metabolites alternative to ethanol, mainly glycerol and pyruvic acid [155,156]. *Candida zemplinina* showed the ability to produce secondary metabolites or volatile compounds by its secreted enzymes including esterases, glycosidases, lipases, β-glucosidases, proteases and cellulases. The protease activity allows this yeast species to improve wine stability by hydrolysing proteins and promoting cellular autolysis. At the same time, its cellulolytic and hemicellulolytic enzymes and glycosidases (β-glucosidase, β-xylosidase, β-apiosidase, α-rhamnosidase and α-arabinofuranosidase) play a key role in promoting the extraction of volatile molecules from the grape berry, releasing terpenes and norisoprenoids during fermentation. *C. zemplinina* species show a strong fructophilic character [156], which is considered a positive fermentative property since limits the production of acetic acid by *S. cerevisiae* during sweet wine production [38]. Moreover, the fructophilic character can be considered as a relevant oenological trait for the selection of non-*Saccharomyces* starter cultures in order to prevent stuck/sluggish fermentation, predominantly in grape must with relevant sugar amount [157]. 

Several studies have recently focused on the employment of *C. zemplinina* in couple with *S. cerevisiae* in co-inoculation or sequential inoculation of must with high sugar contents for the pro special wines such as ‘icewines,’ ‘passito wines’, and ‘botrytised wines’ [38,45,46,156,158]. Englezos et al. [159] reviewed the available data concerning the impact of co-inoculation of *C. zemplinina* and *S. cerevisiae* on wine aroma, showing an increase in higher alcohols, in particular phenylethanol and isoamyl alcohols, esters such as ethyl octanoate and ethyl decanoate, phenylacetate and terpenes like linalool, geraniol and citronellol. These results were confirmed by Russo et al. [45] in a recent research on the impact of mixed starters on the volatile profile of *Negroamaro* wine. It is clear that either in co-inoculation or sequential inoculation, in lab and pilot scale, the coexistence of these species positively affects the aroma of various wines through an increase of ethyl esters and terpenes followed by an increase of higher alcohols, except 2-phenylethanol, and volatile phenols [159]. The fermentation of *Sauvignon Blanc* grape must with of *C. zemplinina* produced a wine denoted by an increased amount of terpenes (citronellol, geraniol, nerolidol and farnesol) and low production of acetate esters (isoamylacetate, hexyl acetate and 2-phenylethyl acetate) [160]. Conversely, higher concentrations of ethyl esters, higher alcohols and short-chain fatty acids were increased in wines produced by co-inoculation of *Macabeo* must with the above-mentioned species, indicating that parameters such as strain compatibility, inoculation protocol and grape must composition affect the volatile profile of the resultant wines. 

Recent studies have investigated the physiological behaviour of enological lactic acid bacteria (LAB) during alcoholic fermentation, promoted by a mixed culture of *C. zemplinina* and *S. cerevisiae.* To highlight these interactions, Russo et al. [158] performed lab-scale vinifications by inoculating grape must with the above yeast species and by promoting malolactic fermentation (MLF) with different *Lactiplantibacillus plantarum* and *Oenococcus oeni* strains, in simultaneous or sequential inoculation. This study showed the peculiar effect of *C. zemplinina* on the evolution of MLF and the significance of strain-dependent interactions with the inoculated LAB. Another recent study evaluated the compatibility and the fermentative performance of mixed starter formulation composed by three autochthonous starter strains belonging to the *S. cerevisiae, C. zemplinina* and *L. plantarum* species, carrying out for the first time pilot- and industrial-scale vinification tests throughout two consecutive vintages [46]. In agreement with previous evidence, the authors found that the wines were characterised by enhanced fruity and floral notes due to an increase of esters and a release of terpenes. It is likely to be explained by the metabolic interactions between yeasts and bacteria [47].

All these results are likely to be the evidence of the changes of in the set of all volatile compounds produced by the yeast (volatome) due to the different combinations of the strains, endorsing the concept that the complexity of the wine can reflect the complexity of the starter cultures [9,53,158].

### 4.6. Pichia spp.

Species belonging to the *Pichia* genus have also been tested in wine fermentation. Wines obtained with *S. cerevisiae* and *Pichia* spp. inoculation were characterised by higher levels of varietal thiols and other volatile compounds, including acetaldehyde, ethyl acetate, 1-propanol, 1-hexanol, *n*-butanol, 2,3-butanediol and ethyl octanoate [48]. Moreover, wines produced by *Pichia* spp. also revealed consistent amounts of polysaccharides, that improved the organoleptic properties of the final product [151]. 

*Pichia kluyveri* is considered a good producer of esters, in particular ethyl octanoate and 2-phenylethyl acetate, and terpenes in sequential fermentations with *S. cerevisiae*. Therefore, it contributes to enhance both the varietal and the fermentation aromas. A study conducted to assess the impact of *P. kluyveri* on the aroma of *Riesling* wine showed a significant increase of the terpenes hotrienol and linalool oxide to concentrations above their odour thresholds [52]. From a sensory point of view, the contribution of *P. kluyveri* to the final bouquet of the produced wine corresponded to an increase in pear and citrus/grape fruit odour notes [52]. *Pichia kluyveri* has shown β-glucosidasic activity, which positively affects the release of terpenes during the fermentation process and the ability to produce higher levels of varietal thiols, especially 3-mercaptohexyl acetate (3MHA) [48]. *P. kluyveri* has been employed as a partner of *S. cerevisiae* to improve the quality of Spanish *Airén* wine produced from neutral grapes [13]. The couple employed showed the best performance in sequential inoculation, allowing an improvement of aroma complexity of the tested wine. Among *Pichia spp*., *P. manshurica*, *P.guilliermondii*, *P.membranifaciens* [49,161] have also been species of oenological interest. Sáez and co-workers [161] highlighted the ability of *P.manshurica* and *P.membranifaciens* to produce volatile phenols in red wine, in particular 4-ethylphenol and 4-ethyl guaiacol. Likewise, Perpetuini et al. [49] observed a high production of volatile phenols exceeding their odour threshold and other molecules responsible for off odour (e.g., 1-propanol 2-methyl, 1-butanol 3-methyl, 1-propanol 3-methylthio, butanoic acid, hexanoic acid, and octanoic acid) when studying the influence of *P. manshurica* on aroma profile of *Montepulciano* must and wine.

## 5. Conclusions

Even though the non-*Saccharomyces* species contribution to wine aroma is the subject of continuous study, this area still needs in-depth analyses. This review summarised the most recent information on selected non-*Saccharomyces* as bioresources to enhance the varietal and fermentative aroma of the wines. The recovery and re-evaluation of non-Saccharomyces yeasts responds to consumers’ need to produce wines with differentiated sensory profiles represented the main target of this review paper. The use of these yeasts could improve wine quality, releasing peculiar metabolites and could positively affect other wine parameters such as alcohol content and acidity. Strains should be selected by taking into account to avoid off-flavours but also to liberate the great aroma potential which characterizes many grapes, in particular the aromatic (e.g., *Muscat* and *Malvasia*) and semi-aromatic (e.g., *Riesling* and *Glera*) grape varieties. This is a topic of interest to improve wine added value, to produce beverages with enhanced regional characteristics, but also of interest to design tailored solutions to cope with modifications in wine characters triggered by global trends such as climate changes. 

## Figures and Tables

**Table 1:** Metabolites produced in wine by non-*Saccharomyces* yeasts in mixed fermentations.

Species	Metabolites	References
*Hanseniaspora* spp.	Acetate esters	[28]
	C6 alcohols	[29]
	Acetic acid	[30,31]
	Sulphur compounds	[32]
		
*Hanseniaspora uvarum*	Phenylethanol	[7]
	Acetate esters and ethyl esters	[7,33]
	Terpens and norisoprenoids	[33,34]
	Acetic acid	[35]
		
*Torulaspora delbrueckii*	Linalool	[36]
	Higher alcohols, esters	[35,37,38]
	Acetate esters	[39]
	Lactones	[40]
	3-Methyltio-1-propanol	[1]
	4-methyl-4-sulfanylpentan-2-one	[41]
		
*Lachancea thermotolerans*	Esters, terpenes	[12,42]
	3-Methylthio-1-propanol	[1]
		
*Metschnikowia pulcherrima*	Terpenes	[32]
	4-Methyl-4-sulfanylpentan-2-one	[43]
	Phenylethanol	[43]
	β-Damascenone	[29]
	Ethyl octanoate, ethyl acetate, 2-phenylethyl acetate	[37,44]
		
*Candida zemplinina*	Higher alcohols	[38,45,46]
	Ethyl esters	[38,45,46,47]
	Terpenes	[48,49,50]
		
*Pichia* spp.	Acetate esters	[51]
	Terpenes	[52]
	3-Mercaptohexyl acetate	[48]
	Volatile phenols	[49,53]
	3-Methylthio-1-propanol	[49]
